# LED Intercanopy Lighting in Blackberry During Spring Improves Yield as a Result of Increased Number of Fruiting Laterals and Has a Positive Carryover Effect on Autumn Yield

**DOI:** 10.3389/fpls.2021.620642

**Published:** 2021-07-27

**Authors:** Anabel Rivas, Kang Liu, Ep Heuvelink

**Affiliations:** Horticulture and Product Physiology, Wageningen University and Research, Wageningen, Netherlands

**Keywords:** supplemental light, intercanopy lighting, blackberries, light emitting diode, bud break, fruiting laterals, fruit quality, yield component analysis

## Abstract

High market price and low availability of local winter and spring production has stimulated production of blackberries in glasshouses at northern latitudes. For this production, light is the main limiting factor. We investigated the potential of intercanopy lighting (ICL) using light emitting diodes (LEDs) to improve blackberry fruit yield in a crop with a spring and an autumn production cycle. During the spring production cycle three light treatments were applied: only natural light (no ICL), 93 or 185 μmol m^–2^ s^–1^ ICL In summer the lateral shoots were cut back and 93 μmol m^–2^ s^–1^ ICL was applied to all plants after cutting back, investigating a possible carryover effect of supplemental light in spring on autumn production. Fresh fruit yield in spring increased by 79 and 122% with 93 and 185 μmol m^–2^ s^–1^ ICL, respectively, compared to no ICL. This represents 3.6 and 2.8% increase in harvestable product for every additional 1% of light. A yield component analysis and leaf photosynthesis measurements were conducted. Maximum photosynthetic capacity (*A*_max_) for leaves at 185 μmol m^–2^ s^–1^ ICL was about 50% higher, and LAI was 41% higher compared to no ICL. ICL increased the number of fruiting laterals per cane, and this explained 75% of the increase in yield. ICL at 185 μmol m^–2^ s^–1^ resulted in a higher yield compared to no ICL, primarily as a result of higher total dry matter production. Furthermore, a higher fraction of dry matter partitioned to the fruits (0.59 compared to 0.52) contributed to yield increase, whereas fruit dry matter content and fruit quality (sugar and acid content) was not affected by ICL. Averaged over the three light treatments autumn yield was 47% lower than spring yield. Autumn yield was 10% higher for plants at ICL 93 μmol m^–2^ s^–1^ in spring and 36% higher for plants at 185 μmol m^–2^ s^–1^ in spring compared to no ICL in spring. This increased autumn yield was caused by more fruiting laterals (less necrotic buds). It is concluded that management practices in spring can have a carryover effect on the autumn production. This is the first scientific paper on the potential for applying LED ICL in blackberries. Further research should focus on optimal intensity of ICL, positioning of supplementary lighting and economic feasibility.

## Introduction

Given the relatively small size of the commercial industry, little work has been done to optimize growth conditions for blackberry (*Rubus* spp.) in glasshouse environments. High market price and low availability of local winter and spring production (Centre for the Promotion of Imports from Developing Countries, 2016) has stimulated production of blackberries in glasshouses at northern latitudes. As reported for other winter-produced crops in northern latitudes, light is a significant environmental factor limiting growth and yield (Marcelis et al., 2006). Consequently, supplemental lighting with High-Pressure Sodium (HPS) lamps during winter has become quite widespread in order to overcome this challenge (Hemming, 2011). Supplemental lighting has been shown to increase photosynthetic rates (Trouwborst et al., 2010) as well as budbreak, for example in roses (Zieslin and Tsujita, 1990). Light-Emitting Diodes (LEDs) make a more energy efficient supplementary lighting possible compared to HPS (Singh et al., 2015). Besides that, LEDs allow for the optimization of light spectra (Massa et al., 2008) and for placing supplementary light in a crop canopy instead of only above the canopy. Higher yields have been reported in cucumber (Hovi et al., 2004) and sweet pepper (Hovi-Pekkanen et al., 2006) when part of the supplementary light is provided as intercanopy lighting (ICL), compared to toplighting only, with the same total supplementary light intensity. These higher yields are mainly due to improved vertical light distribution, which results in increased actual and maximum photosynthesis rates in the lower canopy leaves (Tewolde et al., 2016; Paponov et al., 2020).

Within a canopy receiving only toplighting the exponential decrease in irradiance from the top to the bottom is coupled with a decrease in the red (R): far-red (FR) ratio because unlike red-light (630 nm), the transmission of far-red light (730 nm) through the canopy is quite high (Holmes and Smith, 1977). As has been extensively reported, low red:far-red ratios can cause significant phytochrome-mediated morphological responses (Franklin and Whitelam, 2005) including higher internode elongation, larger leaf expansion, reduced leaf thickness, and reduced branching (Dale and Blom, 2004; Leduc et al., 2014) or reduced budbreak in roses (Mor and Halevy, 1984; Wubs et al., 2014).

Typical glasshouse blackberry production systems make use of dormant, biennial-fruiting cultivars. Unlike tomato or cucumber where the apical meristem is always on top, a blackberry cane has approximately 20–25 potentially active meristems distributed vertically along the cane. After budbreak, subsequent internode elongation and expansion of leaves on the fruiting laterals occurs horizontally, toward the center of the path between rows until a previously formed terminal flower is expressed (Sønsteby and Heide, 2008). In raspberry (*Rubus* spp.), a closely related crop, it has been observed that a lower red:far-red ratio results in higher internode elongation, and therefore longer fruiting laterals in the lower part of the canopy (Sønsteby et al., 2013). In raspberry the uppermost laterals on a shoot tend to produce the fewest inflorescences. Inflorescence complexity in the buds increases along the cane from the top to the base of the main cane, due to a higher number of buds along the inflorescence axis in the lower bud positions (Heide and Sønsteby, 2011). The yield of a blackberry cane is a function of the number of buds along the main cane that produce laterals, as well as the productivity of each of these fruiting laterals. This varies with the percentage of buds within the lateral that express flowers, the quantity of flowers expressed per bud position, and fruit size (Sønsteby et al., 2009). In raspberry, it has been suggested that this yield potential is often not realized due to insufficient light (Fernandez and Pritts, 1994). Therefore, the application of supplemental light in the lower sections of the canopy could not only improve photosynthesis of the lower leaves, but also the morphological development of the meristems and the potential productivity of the fruiting laterals.

In cultivation under high tunnels or rain shelters, it is generally only possible to produce one summer blackberry crop. In greenhouse cultivation, however, the climate can be controlled which creates possibilities for not only increased fruit yield but also two cropping cycles in the same year. It is possible to obtain a second crop cycle (harvest in autumn) by cutting back the fruiting laterals in summer after the spring harvest has stopped (Pitsioudis et al., 2009).

To date, no work has been done on the modification of light quality and light quantity in a blackberry canopy through the use of LED ICL. ICL offers an opportunity to improve the production of blackberry in greenhouses during low-light conditions. Therefore, the objective of this study was to determine the potential of LED ICL for improved blackberry yields, particularly in the lower part of the canopy where production is low. Additionally, this study should improve our understanding of the possible effect higher spring yields as a result of ICL has on autumn yields from the same plants.

Morphological development, growth and yield of blackberry plants under natural light was compared with plants under a low or high intensity of supplemental LED ICL in spring in a greenhouse experiment. After spring harvest, the fruiting laterals were cut back and autumn cycle on the same plants started, with all plants receiving the same amount of ICL. We tested two hypotheses: (1) Blackberry yield per cane in the spring crop will increase under ICL, primarily resulting from a higher number of fruits per lateral, and (2) Applying ICL in spring will improve spring yield at the expense of autumn yield.

## Materials and Methods

### Facilities and Plant Material

On 11 November, 2016, blackberry long cane plants [commercially-grown Driscoll’s variety, interspecific hybrid *Rubus* spp.; nursery located in Abingdon, United Kingdom (51°N, 1°W)] were delivered to Breda, Netherlands. Pots (7 L filled with 100% coir) containing five canes with a minimum cane diameter of 5 mm were selected and placed in cold storage at 2°C. On 11 January 2017, these pots were delivered to Wageningen, Netherlands (52°N, 5.5°E), and placed the following day into two adjacent Venlo-type glasshouse compartments (12 m × 12 m). The canes were pruned to a length of 2.0 m.

In each compartment seven, 9.5 m long plant rows were grown spaced 1.9 m apart, oriented North-South. The distance between pots was 0.38 m. Three adjacent rows were taken as a block. Each block was divided into three plots (three light treatments). Light treatments were allocated according to a latin square and layout was such that buffer rows were kept between the blocks. Each 2.5 m plot contained six pots with the two outer ones as borders on each end. All rows had four buffer pots on each end of the row.

The plants were grown in the glasshouse for almost 1 year. The first (spring) crop cycle took place from 12 January to 7 July, and after cutting back the fruiting laterals a second (autumn) crop cycle took place from 14 July to 22 December.

### Spring Crop Cycle

#### Growing Conditions

Minimum realized temperature during the diel cycle increased gradually from 7°C in February to 12°C in June and July, maximum temperature increased from 17 to 30°C. Liquid CO_2_ was used to enrich the greenhouse air to 600–800 ppm when vents were closed. During ventilation, greenhouse air was kept at ambient CO_2_ level.

Solar radiation was recorded every 5 min. based on a Kipp solarimeter placed outside the glasshouse. Three quantum sensors (Li-190R, Li-Cor Inc., Lincoln, NE, United States) were placed inside each glasshouse compartment, 3.50 m above floor level, near the top of the glasshouse, to measure incoming photosynthetically active radiation (PAR). These sensors were connected to a data logger (Li-1400, Li-Cor Inc., Lincoln, NE, United States). Fraction PAR in solar radiation was assumed to be 0.5 (Jacovides et al., 2004). Greenhouse transmissivity was calculated as the ratio between measured PAR inside the greenhouse and calculated PAR outside.

The fruiting laterals were trellised according to commercial standards. At the onset of flowering, a small hive of bumblebees was introduced in the greenhouse compartments. Two weeks later, the bumblebees were removed and replaced by honey bees. A three stage (vegetative growth, flowering and fruiting) standard blackberry nutrient solution was applied according to commercial standards.

#### LED Intercanopy Lighting Treatments

**FIGURE 1:**
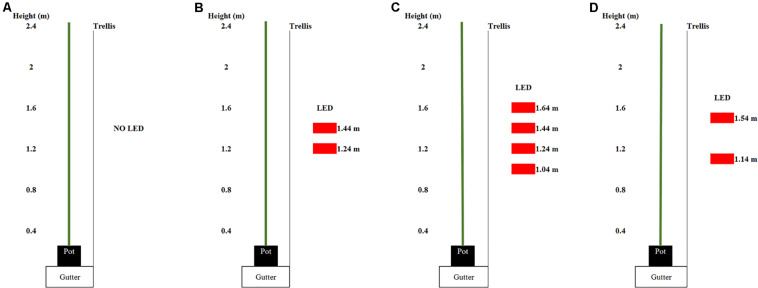
Arrangement of the light emitting diode (LED) modules (displayed as red rectangles) in the spring crop cycle for **(A)** 0, **(B)** 93, and **(C)** 185 intercanopy lighting (ICL) and in the autum crop cycle **(D)**. The numbers beside the LED modules show the distance from the LED module to the ground.

Three light treatments were applied: 0, 93, or 185 μmol m^–2^ s^–1^ ICL. ICL was applied with two (93 μmol m^–2^ s^–1^) or four (185 μmol m^–2^ s^–1^) interlighting LED modules, each 2.5 m long and providing 95% red and 5% blue light (Philips, Greenpower Production Interlighting Module, 107W, 220 μmol s^–1^ PPF, Eindhoven, Netherlands). The total light output of the LED modules has been measured by Philips own certified lab according to IES LM-79-08; CIE S 025/E:2015; prEN13032-4:2013.2 standards. Light intensity at plant level was calculated based on this lamp output and the number of modules per m^2^ ground area. The ICL modules were placed parallel to the row, in the middle of the walkway, 0.95 m from the center trellis. They centered at 1.34 m height relative to the floor and were each spaced 0.20 m apart (Figure 1).

Intercanopy lighting started on 10 February (13 h supplemental light, lamps turned on 1 h before sunrise). From 6 April onward lamps were on only during the natural photoperiod. In order to prevent light pollution between treatments across the blocks, the side of the LED modules facing the buffer rows was covered with aluminum foil. Rectangles of 0.40 by 0.90 m white plastic were centered on the ends of the LED modules and hung on the ends of every plot perpendicular to the row orientation, to prevent light pollution between treatments within a block. These were removed on 22 March once the fruiting laterals were elongated enough such that this light pollution could no longer occur.

#### Destructive Crop Measurements

A total of seven destructive harvests were made over the course of the experiment approximately every 3 weeks from the start of the experiment. The first two destructive harvests were made on two canes randomly selected from the buffer rows (before start of the ICL treatments). The following destructive harvests were made on two canes randomly selected from pots that were not next to each other within the four plant plot, alternating between selected pots across destructive harvest times. Two canes were collected from each treatment in each block (*n* = 6) in all harvests except the last one (11 July) when samples were only collected from one compartment (*n* = 3). When ICL started, cane density was 13.1 canes m^–1^. From the start of fruit harvest until the end of the first crop cycle 3 canes per pot were kept which resulted in 7.9 canes m^–1^.

Bud positions on each cane were numbered from 1 upward, starting at the base of the cane. At each bud position, the fruiting lateral length was measured and the number of receptacles (fruit already harvested), ripe fruits, green fruits, open flowers, closed flower buds, aborted flower buds, and expanded and unexpanded (>1 cm in length) leaves were counted. A lateral was considered elongated when the length from the base to the apex was more than 3 cm. Leaves, fruiting laterals and fruit were grouped in sets of five bud positions: 1–5, 6–10, 11–15, 16–20, and 21+. Measurements including leaf area and dry weight were made in these bud position groups.

All vegetative plant material was dried for 16 h at 105°C in a ventilated oven. Fruit samples were dried at 50°C for 48 h and then at 105°C for 16 h. Primocane shoots (vegetative shoots produced from the crown for production in the following year) were removed, dried, and weighed four times during the first crop cycle.

#### Fresh Fruit Harvest

Fresh fruit was harvested twice a week from 16 May until 4 July. Only fully ripe, black fruit was selected for harvest. Malformed fruit, defined as fruit with more than 50% of the drupelets not fully formed, unevenly ripened fruit and overripe fruit/fruit fallen on the floor were all categorized. Then, all fruit was weighed by category. 10 fully ripe black fruits were randomly selected from the fruit harvested from each plot at every harvest and their fresh weight was determined. These fruits were dried and fruit dry matter content was calculated. This dry matter content was multiplied by the total fresh weight harvested to estimate the total dry weight of the harvested fruit.

#### Fruit Chemical Analysis

Three ripe fruits were collected from each plot from the mid-section of the canopy between 1.04 and 1.64 m on three different dates. Fresh berries were frozen in liquid nitrogen and stored at −20°C. The berries were freeze dried until stable weight was achieved and then powdered, and then dry weight was taken. For extraction of organic acids and carbohydrates, 5 ml of 75% ethanol was added to 12 and 18 mg of powder. Samples were vortexed, put in a water bath (80°C, 20 min) and then vortexed again. Samples were then centrifuged (4°C, 8,790 rpm) and 1 ml of supernatant was pipetted to another tube where ethanol was evaporated out at 55°C. 1 ml deionized water was added, samples were vortexed then put into an ultrasonic bath (10 min). Afterward they were vortexed again and then placed into a centrifuge (10 min). Carbohydrate samples were diluted with deionized water at a ratio of 50:1 and organic acid samples at a ratio of 5:1. Carbohydrate samples were analyzed with HPLC (Thermo Scientific Dionex ICS-5000). Organic acids samples were loaded into a different HPLC (Dionex DX-600).

#### Leaf Photosynthesis

Photosynthesis light-response curves were determined during the week of 6 March which was during vegetative lateral growth, and during the week of 9 May, which was at the onset of fruit harvest. Measurements were conducted on two representative leaves per plot, only for 0 and 185 μmol m^–2^s^–1^ ICL treatments, and in five randomly selected blocks (*n* = 5).

Measurements were conducted using a Li-6400 portable photosynthesis system (LiCor Inc., Lincoln, NE, United States) equipped with a leaf chamber fluorometer (Li-Cor Part No. 6400-40, area 2 cm^2^). During measurements, CO_2_ concentration in the leaf chamber was 400 ppm, the airflow at 400 μmol s^–1^, block temperature at 22°C and RH between 60 and 70%. The percentage of red light and blue light in the chamber was set at 90%/10%. Average achieved leaf temperature across all light steps was higher in the measurements collected in May (26.7 ± 0.47°C) compared to March (21.8 ± 0.24°C) due to issues in the regulation of the block temperature.

Leaves were first adapted to 2,000 μmol m^–2^s^–1^ for approximately 15 min until net photosynthetic rate (*A*_*n*_) and stomatal conductance (*g*_*s*_) were stable. Then data was logged every 5 s, each light step from 2,000 μmol m^–2^s^–1^ down to 1,500, 1,000, 800, 600, 400, 200, 150, 100, 50, 30, and finally 0 μmol m^–2^s^–1^ was held for a minimum of 30 s or until *A*_*n*_ and *g*_*s*_ were stable (Kaiser et al., 2017).

Measured *A*_*n*_ values were averaged over the last 30 s to give one value per light step per leaf. The data for *A*_*n*_ response to absorbed irradiance (assuming 0.85 absorbance of incident light) was then fitted using Equation 1 (Ögren and Evans, 1993).

$$A_{n}=\frac{\varphi *I+A_{\max}-{\left[{\left(\varphi *I+A_{\max}\right)}^{2}-4*\theta *\varphi *I*A_{\max}\right]}^{0.5}}{2^{*}\,\theta }$$

Where φ is the maximal quantum yield, *I* is the absorbed irradiance, *A*_*n*_ is the leaf photosynthetic rate (μmol CO_2_ m^–2^ s^–1^), *A*_max_ is the light-saturated leaf photosynthetic rate and θ is the convexity.

### Autumn Crop Cycle

#### Growing Conditions

On 20 and 21 July, all fruiting laterals from the first crop were cut back to one node below the last fruiting node. On this date, the cane density was 7.9 canes m^–1^ in one experimental compartment and 5.3 canes m^–1^ in the other compartment. This difference occurred from the week of 11 July onward, since a final destructive harvest concluding the spring cycle was taken from one of the compartments only (*n* = 3). On 24 July a destructive harvest was conducted in the other compartment (*n* = 3) such that the cane density during autumn production cycle was 5.3 canes m^–1^ in both compartments. Average greenhouse air temperature gradually decreased from 20°C in July and August to 15°C in November and December. Liquid CO_2_ was used to enrich the greenhouse air to 600–800 ppm when vents were closed. During ventilation, greenhouse air was kept at ambient CO_2_ level.

#### Light Emitting Diode Interlighting Treatments

After the spring cropping cycle, LED modules were rearranged such that all plots had two modules and received 93 μmol m^–2^ s^–1^. In this way supplementary LED light could also be used in the autumn cycle and a possible carry-over effect of spring lighting on autumn production could be studied without the need for more LED modules compared to spring. Modules were placed at a height of 1.14 and 1.54 m (Figure 1). Supplemental lighting (6 a.m. till 8 p.m.; 14 h) started on 28 July.

#### Fresh Fruit Harvest and Destructive Crop Measurements

Fresh fruit was harvested two times per week starting on 3 October until 12 December using the same protocol as for the spring crop. Vegetative laterals that emerged after the July lateral prune were removed once a week. Laterals were considered vegetative if longer than 0.40 m and only having leaves with five leaflets. The laterals were classified by their point of origin within the vertical canopy: 0.0–1.0 m from the floor, 1.0–1.8 m and above 1.8 m. All material was dried and weighed according to the spring protocol.

Two destructive harvests were conducted. The first one was on 24 July, only in the compartment with 7.9 canes m^–1^. Two canes were randomly selected from two pots in each plot (*n* = 3). The second destructive harvest took place at the end of the experiment on 15 Dec. Two canes were selected from each plot in each compartment (*n* = 6).

At each destructive harvest, buds along the cane were numbered as before and data were collected in groups of 10 buds (1–10, 11–20, and 21+). Fruit laterals were categorized as emerging from a primary bud or secondary bud. Secondary buds, axillary buds and scale leaf on each lateral were counted. Additionally, the number of necrotic buds along a fruiting lateral was counted. Buds were considered necrotic when more than 50% of their area was brown. Leaf area, fruiting lateral length, leaf dry weight, fruiting lateral dry weight and cane dry weight of each bud position group previously mentioned were evaluated. On the last destructive harvest date, the number of scars from vegetative laterals as well as the number of unharvested fruits was also counted and weighed. Vegetative and fruit tissues were then oven dried as for the spring cropping cycle.

### Data Analysis

Measures of technical replicates were averaged first and then entered into SPSS (Version 23, IBM Corp, Armonk, NY, United States) as one value for each experimental unit. Analysis of Variance (ANOVA) was conducted to test (*P* = 0.05) for effects of treatment and bud position (when applicable) using row position (*n* = 3), position across greenhouse (*n* = 3) and compartment (*n* = 2) as blocking factors. An error was made during spring fruit harvest in the peak production period, resulting in a missing value for one block, hence final fruit yield analysis was based on five instead of six repetitions. Normality and homogeneity of variance of the residuals was tested using the Shapiro-Wilk test and Levene’s test, respectively. Mean separation was conducted using Fisher’s protected LSD-test at *P* = 0.05. Data that violated the assumptions of normality were transformed using a square root or natural logarithm function. Data that did not fit assumptions of normality after transformation were analyzed using a Kruskal-Wallis test followed by a Mann-Whitney test for mean separation (*P* = 0.05).

## Results

### Spring Crop Cycle

Total light sum incident on the crop was 22 or 45% higher when 93 or 185 μmol m^–2^ s^–1^ ICL was applied, compared to no ICL (Table 1). In February, with an average daily solar light sum of 5.8 mol m^–2^, supplemental light represented 43 or 60% of the total incoming light. In June, the average daily solar light sum was much higher at 30.2 mol m^–2^, therefore supplemental LED light represented only 15 and 27% of the total incoming light for 93 and 185 μmol m^–2^ s^–1^ ICL, respectively.

**TABLE 1:** Natural and supplemental total light sum when no intercanopy lighting (ICL) was applied, or with 93 or 185 μmol m^–2^ s^–1^ ICL, during spring cultivation of blackberries.

	ICL (μmol m^–^^2^ s^–^^1^)		
	0	93	185
Natural light sum (mol m^–2^)	3,342	3,342	3,342
Supplemental light sum (mol m^–2^)	0	755	1501
Total light sum (mol m^–2^)	3,342	4,097	4,843
Light sum increase (%)	–	22.3	44.9

Fruit harvest started mid May for all three treatments. The cumulatively harvested fresh fruit yield per cane was 79 and 122% higher for ICL 93 and 185 μmol m^–2^ s^–1^, respectively, compared to no ICL (Table 2) as a result of more fruits harvested per cane (Table 2). Individual fruit weight was only slightly higher (5%) at 185 μmol m^–2^ s^–1^. Fruit dry matter content was not significantly affected by ICL (Table 2).

**TABLE 2:** Blackberry fresh fruit harvest for 0, 93, or 185 μmol m^–2^ s^–1^ ICL.

Fruit yield parameter (per cane)	ICL (μmol m^–^^2^ s^–^^1^)		
	0	93	185
Marketable fresh fruit weight (g)	636 a^3^	1139 b	1416 c
Non-marketable fresh fruit weight (g)	19.2 a	50.5 b	49.7 b
Number of fruits^1^	61.5 a	111 b	131 c
Fruit weight (g berry^–1^)	10.1 a	10.1 a	10.7 b
Fruit dry matter content (%)^2^	12.0 a	11.9 a	12.1 a

*Fresh fruit was harvested between 16 May and 04 July (Spring production cycle).*

*^1^Square root transformed data used in ANOVA.*

*^2^Log-transformed data used in ANOVA.*

*^3^Different letters within a row indicate significant differences according to Fisher’s protected LSD-test (*P* = 0.05); *n* = 5.*

The number of fruits still on the plant at the end of the spring cropping cycle was higher for both levels of ICL compared to no ICL (Table 3). Vegetative biomass increased with increasing ICL intensity although not statistically significant (Table 3). ICL resulted in a larger fraction of biomass allocated to the fruit (0.59–0.60) compared to no ICL (0.52). For bud positions 11–15, biomass allocation to the fruit was 0.67 for 93 μmol m^–2^ s^–1^ ICL and 0.62 for 185 μmol m^–2^ s^–1^ ICL compared to 0.43 without ICL.

**TABLE 3:** Blackberry crop parameters for 0, 93, or 185 μmol m^–2^ s^–1^ ICL.

Crop parameter (per cane)	ICL (μmol m^–^^2^ s^–^^1^)		
	0	93	185
Total fruit number^1,2^	115 a^4^	185 b	169 b
Number of unharvested (unripe) fruits	45 a	42 a	28 a
Dry weight of unharvested fruit (g)	27.1 a	37.4 a	24.1 a
Increase in cane dry weight (g)	5.07 a	3.69 a	8.89 a
Leaf area (m^2^)	1.32 a	1.69 ab	1.86 b
Leaf dry weight (g)	58.5 a	72.8 ab	91.0 b
Specific leaf area (cm^2^ g^–1^ dry weight)	230 ab	236 b	206 a
Fruiting lateral dry weight (g)	43.1 a	43.4 a	49.0 a
Total vegetative dry weight (g)	102 a	116 a	140 a
Proportion of biomass allocation to fruits (at destructive harvest)^3^	0.52 a	0.60 b	0.59 b

*The final destructive analysis of the plant occurred during the week of 11 July.*

*^1^Square root transformed data used in ANOVA.*

*^2^Includes total number of receptacles (fruit already harvested) and unharvested fruit remaining on plant.*

*^3^Estimated by multiplying number of receptacles, fruit and flower by average dry matter content and fruit fresh weight.*

*^4^Different letters within rows indicate significant difference according to Fisher’s protected LSD-test (*P* = 0.05); *n* = 3.*

**FIGURE 2:**
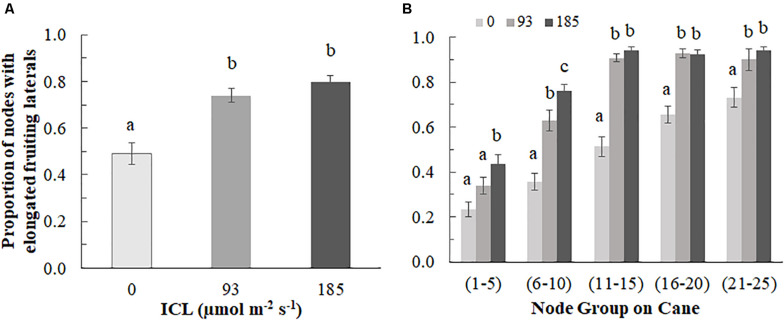
Proportion of buds with elongated fruiting laterals (>3 cm) per blackberry cane for 0, 93, or 185 μmol m**^–^**^2^s**^–^**^1^ ICL **(A)** on the entire cane and **(B)** by bud position group (counting from the base of the cane). Data is an average of observations from destructive harvests on 14 March, 4 April, 24 April, and 11 July. **(A)** Different letters indicate significant differences according to Fisher’s protected LSD-test (*P* = 0.05); **(B)** Different letters within a bud position group, indicate significant differences (Mann-Whitney test, *P* = 0.05). Error bars indicate SEM (*n* = 6).

**FIGURE 3:**
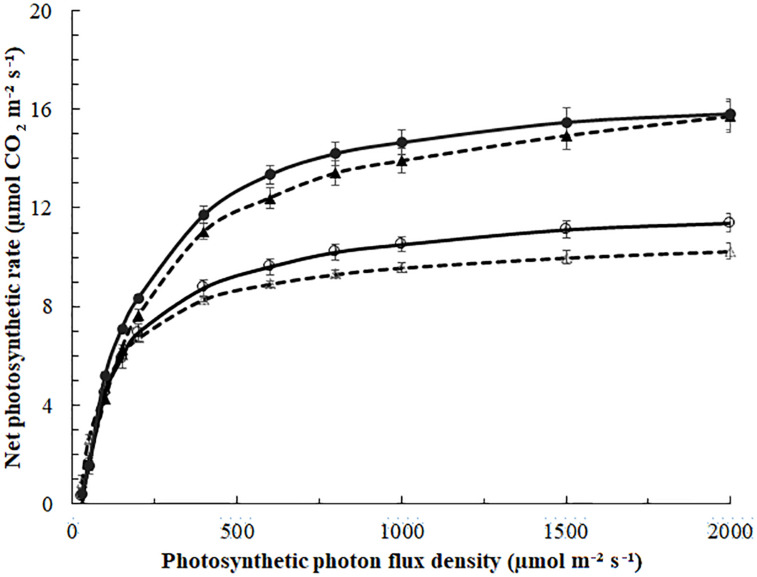
Photosynthesis light-response curves of blackberry leaves exposed to 0 (Δ,⚪) or 185 (▲,⚫) μmol m^–^^2^s^–^^1^ ICL approximately 29 days (March, Δ,▲, - - -) or 89 days (May,⚪,⚫—) after starting the ICL treatment. Leaf temperature was 22°C in March and 27°C in May. Curves represent fitted non-rectangular hyperbola (Eq. 1); parameters in Table 4. Error bars indicate SEM (*n* = 5).

The proportion of elongated laterals was much lower (0.49) on the canes grown without ICL compared to those with ICL (0.74 for 93 μmol m^–2^ s^–1^ and 0.80 for 185 μmol m^–2^ s^–1^) (Figure 2A). ICL of 93 μmol m^–2^ s^–1^ showed a nearly maximum proportion of elongated laterals in bud positions 11–15, which did not change when 185 μmol m^–2^ s^–1^ ICL was applied (Figure 2B). In bud positions 6–10, 93 μmol m^–2^ s^–1^ ICL increased the proportion of elongated laterals by 76%, while 185 μmol m^–2^ s^–1^ ICL resulted in a 113% increase compared to no ICL. ICL treatments did not significantly influence total fruit number per fruiting lateral (Supplementary Table 1). The correlation between fruit fresh yield per cane and number of fruiting laterals per cane was 0.867 (Figure 3), which means that 75% of the variation in yield per cane was explained by the number of fruiting laterals per cane.

**TABLE 4:** Photosynthesis light-response curve (Eq. 1) parameters for blackberry leaves grown at 0 or 185 μmol m**^–^**^2^ s**^–^**^1^ LED ICL: quantum yield (φ; μmol μmol**^–^**^1^), convexity (θ), and maximum assimilation rate (*A*_max_; μmol CO_2_ m**^–^**^2^ s**^–^**^1^).

Month	Photosynthetic parameter	Intercanopy lighting (μmol m^–^^2^ s^–^^1^)	
		0	185
**March**			
	φ	0.07 a^1^	0.06 b
	θ	0.54 a	0.63 a
	*A*_max_	10.5 a	16.5 b
**May**			
	φ	0.06 a	0.06 a
	θ	0.67 a	0.75 a
	*A*_max_	11.6 a	16.4 b

*^1^Different letters within rows indicate significant difference according to Fisher’s protected LSD-test (*P* = 0.05); *n* = 5.*

**FIGURE 4:**
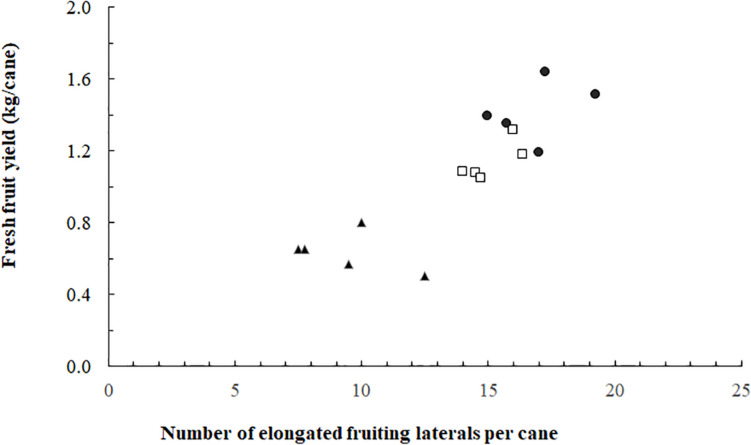
Blackberry fresh fruit yield per cane as a function of the number of fruiting laterals per cane (spring crop cycle). Data for 0 (▲), 93 (◻), and 185 (⚫) mol m^–2^ s^–1^ ICL (*n* = 15; five replicates for each of the three ICL treatments).

In March, the quantum yield, or initial slope of the photosynthesis light-response curve (Figure 4) was higher in leaves of no ICL compared to leaves exposed to 185 μmol m^–2^ s^–1^ ICL (Table 4). This difference between treatments had disappeared in May. In March, *A*_max_ for 185 μmol m^–2^ s^–1^ ICL was a 58% higher compared to no ICL. This difference was somewhat lower in May (42%) caused by a higher *A*_*m*__*ax*_ for no ICL.

**FIGURE 5:**
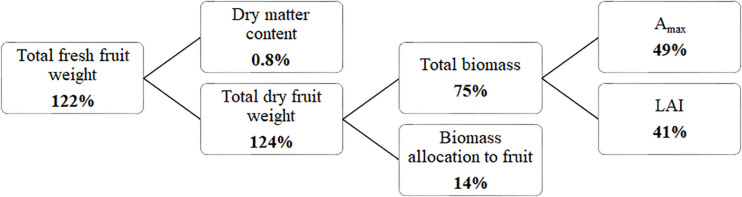
Yield component analysis for a blackberry crop (spring crop cycle). Percentages indicate how much higher the component was for 185 μmol m^–2^ s^–1^ ICL compared to no ICL. Yield (kg fresh fruit mass m^–2^) = Total dry mass (kg plant dry mass m^–2^) × Fraction to fruits (fruit dry mass/total dry mass)/fruit dry matter content (fruit dry mass/fruit fresh mass).

Considering fruit fresh yield as the product of total dry matter production and fraction of dry matter partitioned to the fruits divided by the fruit dry matter content revealed (Figure 5) that 185 μmol m^–2^ s^–1^ ICL resulted in a higher yield compared to no ICL, primarily as a result of higher total dry matter production. Besides more light, a higher LAI and *A*_max_ contributed to this higher dry matter. Furthermore, a higher fraction of dry matter partitioned to the fruits contributed to yield increase, whereas fruit dry matter content was not influenced by ICL.

Intercanopy lighting did not influence glucose, fructose, sucrose, malate, citrate, and isocitrate concentrations in the fruit (Table 5).

**TABLE 5:** Sugar and organic acids content of ripe blackberry fruits grown at 0, 93, or 185 μmol m^–2^ s^–1^ ICL.

Sugar/acid (g/kg fresh)	ICL (μmol m^–^^2^ s^–^^1^)		
	0	93	185
Glucose	38.5 a^1^	39.6 a	38.4 a
Fructose	37.4 a	38.5 a	37.4 a
Sucrose	7.0 a	6.5 a	6.9 a
Malate	1.5 a	1.6 a	1.7 a
Citrate	0.10 a	0.10 a	0.10 a
Isocitrate	8.3 a	7.7 a	7.2 a

*^1^Different letters within rows indicate significant difference according to Fisher’s protected LSD-test (*P* = 0.05); *n* = 6.*

### Autumn Crop Cycle

Despite receiving identical amounts of light during the autumn crop cycle, yield of plants that received 93 or 185 μmol m^–2^ s^–1^ ICL in spring were 11 and 36% higher compared to no ICL in spring, respectively. Only for 185 μmol m^–2^ s^–1^ ICL this increase in fresh and dry fruit yield was statistically significant (Table 6). Just like in spring, the higher fruit yield resulted from a larger number of fruit harvested, not from heavier fruit (Table 6). The total number of fruit remaining on the plant when harvest stopped was not significantly affected by spring ICL intensities. Total fruit number per cane was significantly higher for 185 μmol m^–2^ s^–1^ ICL applied in spring, compared to no ICL in spring (Table 6). Although not statistically significant, 93 μmol m^–2^ s^–1^ ICL during spring, resulted in 27% higher total fruit number. Number of harvested ripe fruit was about 56% of the total fruit number and the fruit number remaining on the plant accounted for about 30%. Total fruit number was about 3 times higher for bud positions 11 to 20 compared to lower or higher bud position groups (Supplementary Table 2). Averaged over the 3 ICL treatments autumn yield was 47% lower than spring yield. No ICL during the spring cycle resulted in the highest amount of necrotic buds per cane at the start of the autumn production cycle, whereas the total lateral shoot length did not significantly differ (Table 7).

**TABLE 6:** Blackberry yield per cane during the autumn production cycle (harvest from 3 October till 12 December as affected by spring production cycle ICL treatments.

	ICL intensity (μmol m^–^^2^ s^–^^1^)		
	0	93	185
**Marketable**			
Fruit fresh weight (g)	488 a^1^	540 a	664 b
Harvested fruit number	57.9 a	70.0 b	84.6 c
Fruit dry weight (g)	55.1 a	62.1 a	76.1 b
**Non-marketable**			
Fruit fresh weight (g)	48.9 a	73.5 b	102.7 c
Remaining fruit number^2^	29.1 a	38.5 a	41.7 a

*^1^Different letters in a row indicate significant differences according to Fisher’s protected LSD-test (*P* = 0.05).*

*^2^The remaining fruit harvest includes unripe fruit and was conducted on Dec 14th.*

**TABLE 7:** Total number of necrotic buds and total lateral length (destructive measurement at start of autumn cycle, 24–28 July). No significant interaction between bud position and spring ICL intensity was found.

	ICL (μmol m^–^^2^ s^–^^1^)			Bud position^3^		
Parameter (per cane)	0	93	185	1–10	11–20	21+
Number of necrotic buds ^1^	13.3 b^2^	10.0 a	9.2 a	11.4 a	17.8 b	3.3 c
Total lateral length (cm)	325 a	390 a	398 a	126 a	192 b	54 c

*^1^Buds with internal brown colour larger than 50% of their area were considered necrotic.*

*^2^Means followed by a different letter in a row comparing the 3 ICL intensities, or comparing the 3 bud positions differ significantly according to Fisher’s protected LSD-test (*P* = 0.05), *n* = 3.*

*^3^Bud position counted from the base of the cane.*

## Discussion

### Light Emitting Diode Intercanopy Lighting Increased Number of Fruiting Laterals and Therefore Yield

Supplementary light has been shown to increase yield in several crops like raspberry (Carew et al., 2003; Sønsteby and Heide, 2008), tomato (Lu et al., 2012), and cucumber (Hao and Papadopoulos, 1999). Intercanopy lighting at 93 or 185 μmol m^–2^ s^–1^ increased blackberry fresh fruit yield by 79 and 122%, respectively, compared to no ICL (Table 2). This represents 3.6 and 2.8% increase in harvestable product for every additional 1% of light (Table 1). This increase in yield is much larger than the often-cited rule of thumb of 1% yield increase resulting from 1% more light (Marcelis et al., 2006). This effect might be somewhat overestimated, as the number of unripe fruit, removed when cutting back for the autumn production cycle, was much higher without ICL (Table 3). At 185 μmol m^–2^ s^–1^ ICL 17% of the fruit was left unharvested, whereas without ICL this was 39%. These unripe fruits would have been harvested ripe when spring cycle was continued for a few more weeks. Taking this into account, based on fruit numbers, for 185 μmol m^–2^ s^–1^ ICL yield would have increased by exactly 1% for every additional 1% of light. However, a delay in cutting back would have negatively affected autumn production. Fruit sugar and carbohydrate contents were not significantly affected by ICL (Table 5). Similarly, in tomato increase in yield was reported with LED supplemental lighting while sugar (Lu et al., 2012) and soluble solid (Paponov et al., 2020) content remained unchanged. It is commercially of great importance that such a large yield increase was obtained without negative impact on some key flavor components.

We hypothesized that the number of fruit per lateral would increase when ICL was applied, however, there appeared to be no significant treatment effect on this yield component (Supplementary Table 1). Instead, we observed that the yield component most affected by ICL was the number of elongated fruiting laterals. Approximately 49% of the laterals elongated when no ICL was applied, whereas this was 74% for 93 μmol m^–2^ s^–1^ ICL and slightly higher for 185 μmol m^–2^ s^–1^ ICL (Figure 2A). Yield increases in roses in response to supplementary lighting applied in winter have also been attributed to increased bud break (Khosh-Khui and George, 1977). ICL with LEDs (^∗^0% red and 20% blue) increased the red:far-red ratio at the middle and low positions in a tomato canopy (Paponov et al., 2020), which is known to stimulate bud break (Wubs et al., 2014). Our experiment does not allow to discriminate between the effect of light intensity and light quality on bud break. However, Wubs et al. (2014) concluded that local light intensity, not red:far-red ratio, was the most important factor influencing bud break in rose.

The proportion of elongated laterals in biennial-producing canes of raspberries tends to be higher in the top of the cane due to paradormancy imposed on the basal buds by the apical buds (White et al., 1999). In this experiment, a similar response was observed. Consequently, from bud position 11 and higher, 93 μmol m^–2^ s^–1^ ICL seemed to provide enough light to achieve maximum number of elongated fruiting laterals (Figure 2B) as 185 μmol m^–2^ s^–1^ ICL did not further increase this number. For the basal buds (position 1–10), the fruiting lateral elongation showed a positive correlation with increasing light intensity. In rose, the role of local light on the bud (Girault et al., 2008; Roman et al., 2016), has been shown to function mainly by influencing the ability of the developing shoots to draw assimilates (Mor and Halevy, 1980). The unsaturated response to ICL intensities for the basal buds (position 1–10) suggests further increases in lateral elongation rates could have been achieved by supplying higher light intensities directly on the lower part of the canes.

### Biomass Partitioning and Leaf Photosynthesis

Biomass partitioning to the fruits was higher when ICL was applied (Table 3), which is in accordance with various works reported for other crops (Marcelis, 1993). The largest positive effects of ICL were found in bud positions 11–15, the positions receiving the highest supplemental light intensity and red:far-red ratio (data not shown). Biomass partitioning to the fruit is highly correlated with the number of fruits (Marcelis, 1996) and we observed the greatest increases in fruit number under ICL in the lower part of the canopy. The low SLA at 185 μmol m^–2^ s^–1^ ICL reflects thicker leaves (Table 3) and is a well-known acclimation response to higher light intensity (Evans and Poorter, 2001).

Under low light conditions, quantum yield and convexity of the photosynthesis light-response curve are the most important parameters for assessing the productivity of a leaf. In March, leaves not exposed to ICL showed a higher quantum yield compared to 185 μmol m^–2^ s^–1^ ICL (Table 4), which suggests higher efficiency under low light conditions (Boardman, 1977). In May, this difference had disappeared. In both March and May, leaves exposed to 185 μmol m^–2^ s^–1^ ICL showed a higher *A*_max_, 58 and 42%, respectively, when compared to no ICL. ICL resulting in increased leaf photosynthetic capacity for lower leaves in the canopy was also shown by Dueck et al. (2012) for tomato and Pettersen et al. (2010) for cucumber.

### Positive Effect of ICL on Yield in Spring Carries Over to Autumn Production Cycle

We hypothesized that an increased spring production as a result of spring ICL would come at the expense of autumn production. However, results showed the opposite, ICL in spring increased spring production (Table 2) as well as autumn production (Table 6). The number of secondary laterals from scale buds increased with increased supplementary light intensity in spring (Table 4). This increase is most likely caused by the lower number of necrotic buds. A negative relationship between light intensity and bud necrosis was also found in grapevines. A period of 15 days of shading of individual buds or entire shoots at photosynthetic photon flux density (PPFD) less than 1–2% of full sunlight was sufficient to significantly raise bud necrosis above that of non-shaded control vines (Perez and Kliewer, 1990).

The much lower yield in the autumn cycle compared to the spring cycle probably results from the lower light levels during the fruit production period in autumn.

This it the first scientific report on the potential for applying LED ICL in greenhouse-grown blackberries. Further research should focus on optimal intensity of ICL and the positioning of supplementary lighting (position of ICL modules in the crop, but also ratio between toplighting and ICL). Furthermore, a yield increase by 79% in spring production and a positive carryover effect of 11% yield increase in autumn as a result of 93 μmol m^–2^ s^–1^ ICL is very promising and certainly warrants investigation in the economic feasibility of ICL in blackberries.

## Conclusion

• Applying 93 or 185 μmol m^–2^ s^–1^ ICL in greenhouse-grown blackberry during spring increased spring fresh fruit yields by 79 and 122%, respectively.
• Higher yield mainly resulted from higher total dry matter production and to a lesser extend from a higher partitioning to the fruits.
• A larger number of elongated laterals per cane under LED ICL explained 75% of this yield increase.
• Fruit sugar or acid content was not influenced by LED ICL.
• Autumn yield was 11% higher for 93 μmol m^–2^ s^–1^ and 36% higher for 185 μmol m^–2^ s^–1^ spring ICL, despite the fact that in autumn no difference in LED light treatments was implied (same light level).
• This increased autumn yield was caused by more fruiting laterals (less necrotic buds).

## Data Availability Statement

The raw data supporting the conclusions of this article will be made available by the authors, without undue reservation.

## Author Contributions

AR and EH designed and initiated the experiment. AR conducted the measurements and analysis for the spring cropping cycle and wrote a first draft of the manuscript. KL conducted the measurements and analysis for the autumn cropping cycle. EH supervised the measurements and data analysis and finalized the manuscript. The manuscript has been read and approved by all authors.

## Conflict of Interest

The authors declare that the research was conducted in the absence of any commercial or financial relationships that could be construed as a potential conflict of interest.

## Publisher’s Note

All claims expressed in this article are solely those of the authors and do not necessarily represent those of their affiliated organizations, or those of the publisher, the editors and the reviewers. Any product that may be evaluated in this article, or claim that may be made by its manufacturer, is not guaranteed or endorsed by the publisher.
