# Supplementary material for: LED Intercanopy Lighting in Blackberry During Spring Improves Yield as a Result of Increased Number of Fruiting Laterals and Has a Positive Carryover Effect on Autumn Yield
Source: Front Plant Sci. 2021 Jul 27;12:620642. doi: 10.3389/fpls.2021.620642 (PMC8354201; doi:10.3389/fpls.2021.620642)
Supplement: Supplementary file 1 [file Data_Sheet_1.PDF]

Supplementary Material

Table S1. Number of fruit[1] per lateral within a bud position group for blackberry grown with 0, 93 or 185 μmol m^{-2} s^{-1} intercanopy lighting (ICL) at the final destructive harvest on 11 July.

	Intercanopy lighting (ICL) μmol m^{-2} s^{-1}		
Bud position group	0	93	185
(21+)	22.9 a[2]	21.1 a	14.2 a
(16-20)	9.1 a	9.1 a	8.6 a
(11-15)	6.8 a	7.9 a	8.0 a
(6-10)	7.4 a	8.2 a	6.3 a
(1-5)	7.0 a	3.0 a	8.9 a

[1]Total fruit number was calculated by summing the total number of fruiting structures per fruiting lateral including: receptacles, black fruit, red fruit, green fruit, open flowers and closed flower buds.

[2]Different letters within a row indicate significant difference according to Fisher's protected LSD-test (P = 0.05); n=3.

Table S2. Total fruit number[1] per cane in the autumn blackberry crop cycle, as affected by spring production cycle ICL treatments. No significant interaction between bud position group and light treatment was observed.

	ICL intensity (μmol m^{-2} s^{-1})			
Bud position group	0	93	185	Mean[2]
1 to 10	18.4	23.8	35.7	25.9 a
11 to 20	56.6	75.1	95.8	75.8 b
21+	25.3	25.8	24.8	25.3 a

Total[2]	100.3 a	124.7 ab	156.3 b

[1]Total fruit number was observed at final destructive harvest by summing the total number of fruiting structures per fruiting lateral including: receptacles, black fruit, red fruit, green fruit, open flowers and closed flower buds.

[2]Means or totals followed by different letters differ significantly according to Fisher's protected LSD-test (P=0.05), LSD=13.0 for comparing bud position groups, LSD = 51.9 for comparing ICL intensities.
